# Suprasellar desmoplastic infantile astrocytoma and ganglioglioma: an institutional series report and a clinical summary of this rare tumor

**DOI:** 10.3389/fonc.2025.1674829

**Published:** 2025-12-10

**Authors:** Qiguang Wang, Yi Zhang, Huan Zhang, Xuhui Hui, Wenke Liu

**Affiliations:** 1 Department of Neurosurgery, West China Hospital, Sichuan University, Chengdu, Sichuan, China; 2 Research Core Facility of West China Hospital, Sichuan University, Chengdu, Sichuan, China; 3 Department of Neurosurgery, The Affiliated Chengdu 363 Hospital of Southwest Medical University, Chengdu, Sichuan, China

**Keywords:** suprasellar DIA/DIG, clinical features, differential diagnosis, treatment, outcome

## Abstract

**Background:**

Desmoplastic infantile astrocytomas/ganglioglioma (DIA/DIG) arising in the suprasellar region are extremely rare, and their clinical features, optimal management, and outcomes remain unclear. We aimed to summarize the clinical manifestations, treatment strategies, and prognosis of this rare tumor entity.

**Patients and methods:**

This retrospective case series included 5 institutional cases and 13 literature cases of suprasellar DIA/DIGs. The clinical and radiological characteristics, therapies, and outcomes of this rare tumor were examined.

**Results:**

Our case series comprised 2 males and 3 females, with a median age of 6 months. Three patients underwent partial resection, and 2 had subtotal resection. During follow-up, the residual tumors in 2 patients showed spontaneous regression, 1 tumor progressed, and 2 remained stable. The literature review, including our cases, yielded 18 suprasellar DIA/DIG. Of these, 10 were male and 8 were female, with a median age of 4 months (range, 1–85 months). Ten patients had solitary suprasellar tumors and 8 had multifocal lesions. Over a median follow-up of 25 months, tumor progression of the suprasellar lesions was seen in 9 patients. Notably, spontaneous regression of the suprasellar tumors occurred in 2 patients.

**Conclusions:**

Despite being WHO grade I, suprasellar DIA/DIG can demonstrate multifocal CNS disease and high risk of progression after subtotal resection. DIA/DIG should be considered in the differential diagnosis of suprasellar lesions in infants and long-term close monitoring is warranted.

## Introduction

Desmoplastic infantile astrocytoma/ganglioglioma (DIA/DIG) are rare brain tumors that typically present within the first two years of life (1–4). In the last 2021 World Health Organization (WHO) classification of brain tumors, DIA and DIG are classified together as low-grade (Grade 1) neoplasms in the group of “neuronal and glioneuronal tumors” (4). They are clinically characterized by progressive signs of increased intracranial pressure and rapidly enlarging head circumference (2, 5). On MRI, DIAs/DIG usually appear as solitary, cortical-surfacing, solid-cystic supratentorial neoplasms (6). Previous studies have found that patients with hemispheric DIAs/DIG generally have favorable outcomes with long disease-free survival after gross total resection (5, 7–9). However, the prognosis of suprasellar DIAs/DIG is poor (1, 2). From the review of the pertinent literature, Bianchi et al. reported that all mortality cases of DIA/DIG (6 of 107 cases) involved tumors located in the suprasellar region (1).

Since suprasellar DIA/DIG are extremely rare, with only 13 cases reported in the literature, their clinical features, optimal management, and outcomes remain unclear. Here, we present a retrospective case series of 5 patients with suprasellar DIA/DIG from our institution, and review the 13 other reported cases. Our goal is to summarize the clinical characteristics of this rare entity, elucidate optimal treatment strategies, and determine overall prognosis.

## Patients and methods

### Case series

This retrospective study evaluated patients with histologically confirmed suprasellar DIA/DIG (WHO grade I) who underwent surgical resection at the Department of Neurosurgery, West China Hospital from June 2009 to October 2019. Clinicopathological details including age, sex, clinical presentations, radiological features, treatment methods, and clinical outcomes were reviewed. The study was approved by the Institutional Review Board of West China Hospital.

All patients underwent preoperative cranial and spinal MRI with intravenous contrast. Imaging findings such as tumor texture (solid or cystic), size, number of lesions (single or multifocal), enhancement pattern, and peritumoral edema were evaluated. Surgical extent was determined by reviewing postoperative MRI scans. Gross total resection was defined as complete resection, subtotal resection as <10% remnant, and partial resection as ≥10% remnant. Histopathological examination included standard H&E staining, and immunohistochemistry for BRAF-V600E, IDH1-R132H, GFAP, synaptophysin, neurofilament protein, S100, Ki-67, and olig-2. Follow-up occurred at 3 months, 6 months, and annually after surgery. Time of death was recorded as final follow-up for deceased patients.

### Literature review

We conducted a literature review of suprasellar DIAs/DIGs per the PRISMA (preferred reporting items for systematic reviews and meta-analyses) guidelines (10). We searched the PubMed/Medline database to collect the literature data up to October 2019 without early date limit, using the keyword “desmoplastic infantile gangliogliomas” or “desmoplastic infantile astrocytomas”. The following search algorithm was applied in the Pubmed/MEDLINE database: (“Desmoplastic Infantile Astrocytoma”[Title/Abstract] OR “Desmoplastic Infantile Ganglioglioma”[Title/Abstract] OR “Desmoplastic Infantile Tumors”[Title/Abstract] OR “DIT”[Title/Abstract] OR “DIA”[Title/Abstract] OR “DIG”[Title/Abstract]) AND (Case Reports [Publication Type] OR “Case Series”[Title/Abstract] OR “Clinical Data”[Title/Abstract]). We included only publications written in English and enrolled patients of all ages, sexes, and ethnic backgrounds. Eligible studies comprised case reports, case series, and prospective or retrospective analyses in which the diagnosis of DIA/DIG was histologically confirmed. Reports lacking a definitive diagnosis or sufficient histopathological evidence for DIA/DIG were excluded from the review. Preclinical investigations, narrative reviews, editorials, and correspondence pieces such as letters to the editor were excluded from the analysis. Duplicate articles were removed. Two investigators (Q.G. and W.K.) independently screened the titles and abstracts of all retrieved articles. Studies deemed potentially relevant were then assessed in full text to determine eligibility. Discrepancies between the two reviewers were resolved through discussion and consensus with a third investigator (H.Z.). All case reports and case series were carefully reviewed and only cases with tumors in the suprasellar region were included in this study. Next, we extracted each patient’s demographic, clinical, radiological and histopathological features, treatment (extent of surgery, postoperative adjuvant therapy) and outcome data. Furthermore, we conducted a comparison of clinical features between single suprasellar DIA/DIG and suprasellar DIA/DIG with multifocal lesions.

## Results

### Patient characteristics

Our institutional review identified 5 patients (2 males, 3 females) with suprasellar DIA/DIG, with a median age of 6 months (range 1–85 months). Presenting symptoms included seizures (n=2), nystagmus (n=2), and downward gaze (n=1). Baseline were summarized in **Table 1**.

**Table 1:** Description of the five patients with suprasellar DIA/DIGs.

Case. no	Age(months)	Sex(M/F)	Symptoms	Location	Texture	Radiological features				Treatment	Follow-up (months)	Recurrence/ progression	Outcome
						T1‐weighted image (WI)	T2‐weighted image (WI)	Enhancement	Edema				
1	85	Female	Nystagmus	Single	Solid	Hypointense	Isointense	Heterogeneous	No	STR+CTR	60	Stable	Alive
2	6	Male	Nystagmus	Multifocal	Solid	Isointense	Isointense	Heterogeneous	Obvious	PTR	26	Regression	Alive
3	12	Male	Downward gaze gaze	Single	Solid	Hypointense	Hyperintense	Homogeneous	No	PTR	15	Progressed	Dead
4	1	Female	Seizures	Single	Solid	Isointense	Isointense	Heterogeneous	Mild	STR	24	Stable	Alive
5	2	Female	Seizures	Single	Solid	Isointense	Isointense	Heterogeneous	No	PTR	41	Regression	Alive

### Radiological manifestation

**Figure 1:**
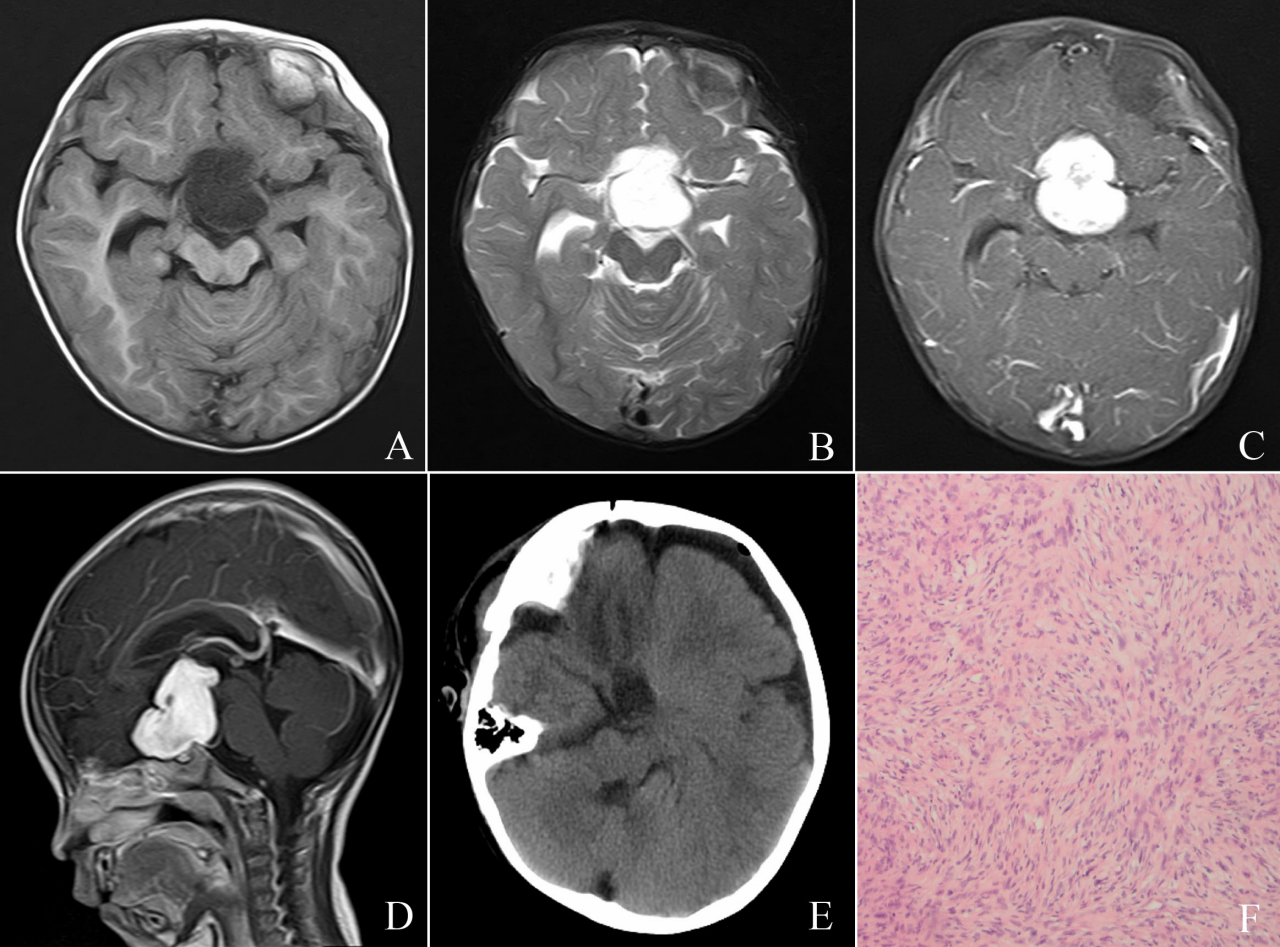
Cranial MR images **(A-D)**: Preoperative cranial MRI demonstrated a suprasellar solid mass in a 12-month-old male, with hypointense on T1-weighted sequences **(A)** and hyperintense on T2-weighted image **(B)**; Gd-enhanced MRI depicted homogeneous enhancement **(C, D)**. The patient underwent partial tumor resection **(E)** and postoperative histological examination supported the diagnosis of DIA/DIG **(F)**.

Gd-enhanced MRI was performed in all patients. The MRI characteristics were shown in **Table 1**. Four patients had solitary suprasellar tumors, while case 2 showed multifocal lesions in the suprasellar cistern, bilateral cavernous sinus and hippocampus, intraorbital and cisterna magna. On MRI, suprasellar DIA/DIGs appeared hypointense or isointense on T1, hyperintense or isointense on T2, and heterogeneously/homogeneously enhancing after contrast (**Figure 1**). Peritumoral edema was seen in 2 patients.

### Treatment and histopathology

All patients underwent surgical resection in our institution, 2 subtotal and 3 partial; we used 3D-slicer software (4.11 version) to measure the preoperative and postoperative tumor volume. One patient received adjuvant oral temozolomide; no patient received radiotherapy. Histological examination showed that the tumor consisted of desmoplastic spindle cells with densely eosinophilic collagenous, without identifiable differences with the hemispheric compartments (1). Immunohistochemistry was positive for GFAP, S-100, and Olig-2; negative for Syn and Neu-N. The Ki67 index was 5% in 3 cases. BRAF V600E mutation was detected in case 2; cases 4 and 5 were wild-type. Four tumors were IDH1-R132H-negative (**Table 2**).

**Table 2:** Immunohistochemistry characterization of suprasellar DIA/DIGs.

Case no.	IDH1-R132H	BRAF-V600E	GFAP	Syn	Neu-N	S-100	Ki-67	Olig-2
1	Negative	NA	Positive	Positive	Positive	Positive	NA	Positive
2	Negative	Positive	Positive	NA	Negative	Positive	5%	Positive
3	Negative	NA	Positive	Negative	Negative	Positive	5%	Positive
4	NA	Negative	Positive	Negative	Negative	Positive	5%	Positive
5	Negative	Negative	Positive	Negative	Negative	Positive	NA	Positive

### Clinical outcomes

During the median follow-up of 25 months, spontaneous regression of suprasellar lesions occurred in 2 patients. One patient showed progression, 2 had stable residual tumors. At the last follow-up, patient 3 died from malignant intracranial hypertension due to tumor progression and acute hydrocephalus. Patients 1 and 4 had visual deficits, while the other two patients were alive without additional symptoms.

### Literature review

Our literature review identified 13 additional reported cases of suprasellar DIA/DIG. In total, 18 cases (including our series) comprise the current data on this entity (**Supplementary Table 1**). The baseline data was demonstrated in **Table 3**. Of these, 10 were male and 8 were female, with a median age of 4 months (range 1–85 months). The most common presenting symptoms were macrocephaly (7/18), nystagmus (6/18), seizures (4/18), and signs of increased intracranial pressure (3/18). Ten patients had solitary suprasellar lesions, while 8 had multifocal tumors involving the cerebellar hemisphere, ventricles, posterior fossa, and spinal cord. Regarding texture, 14 were solid and 4 were solid-cystic. Sixteen patients underwent surgery (6 subtotal, 10 partial resections/biopsies); 2 were managed expectantly. Eight received postoperative chemotherapy.

**Table 3:** Patients characteristics.

Patient characteristics	Number (%)
Cases	18
Gender	
Male	10
Female	8(44.4%)
Age, months (median)	4
Range	1-85
Number of lesions	
Single	10(55.6%)
Multiple	8(44.4%)
Tumor texture	
Solid	14(77.8%)
Solid-cystic	4(22.2%)
Extent of surgery (Suprasellar lesion)	
Subtotal	6(33.3%)
Partial/Biopsy	10(55.6%)
Observation	2(11.1%)
Postoperative chemotherapy	8(44.4%)
Tumor progression (Suprasellar lesion)	9(50%)
Tumor regression (Suprasellar lesion)	2(5.6%)
Follow-up period	
Months (median)	25
Range	0.25-139
Outcome	
Dead	5(27.8%)
Alive	13(72.2%)
Symptoms	
Macrocephaly	7(38.9%)
Headache, vomiting, increased intracranial ICP	3(16.7%)
Seizures	4(22.2%)
Nystagmus	6(33.3%)
Others	1(5.6%)

During a median follow-up of 25 months, progression of the suprasellar lesion occurred in 9 patients. Notably, 2 patients showed spontaneous regression. Five patients died from progression/panhypopituitarism (2), 1 from malignant hypertension (11), 1 from progression (12), 1 from hypothalamic dysfunction (13), and 1 in our study from malignant intracranial hypertension.

## Discussion

DIA/DIG are rare pediatric brain tumors, comprising 0.1-1.25% of all cases (2). They typically present under 18 months of age (14) and are most commonly located in the supratentorial temporal, frontal, and parietal lobes (15–17). Suprasellar DIA/DIG are extremely rare; details regarding clinical features, optimal management, and outcomes remain unclear. Our study aimed to summarize the characteristics of suprasellar DIA/DIG and elucidate optimal treatment strategies for this rare entity.

In contrast to supratentorial hemispheric DIA/DIG, which typically demonstrate a cystic component with cortical-based enhancement on MRI (1), suprasellar DIA/DIG often appear as homogenously enhancing lesions without cystic areas. They frequently exhibit isointense or hypointense T1 signal, isointense or hyperintense T2 signal, and strong enhancement on contrast MRI. While hemispheric DIA/DIG are usually solitary, 44.4% of suprasellar lesions were multifocal in our study. Besides the suprasellar region, additional tumor locations included the cerebral lobes, ventricles, posterior fossa, and spinal cord – all sites within the CSF pathway. Setty et al. reported that CSF collected from ventricles contained tumor cells (18), Darwish et al. reported that a suprasellar DIA/DIG depicted tumor metastases in basal cisterns, leptomeningeal and subarachnoid space six weeks after initial surgery (13). Therefore, suprasellar DIA/DIG may represent a more aggressive phenotype with potential for CSF-mediated tumor spread. In summary, DIA/DIG should be included in the differential when encountering suprasellar masses or multifocal CNS lesions in infants/young children. Careful long-term monitoring for disease progression is warranted.

Gross total resection is the standard treatment for DIA/DIG and confers a favorable prognosis (3, 17, 19). However, we found suprasellar DIA/DIG frequently had strict adhesion to the surrounding tissue and hyper-vascularized structure intraoperatively, and the proximity of suprasellar DIA/DIG to critical brain structures such as optic nerve and chiasm, hypothalamus, pituitary gland and infundibulum make total resection more difficult and often impossible. In present study, six patients underwent subtotal resection, ten patients underwent got partial resection or biopsy, and the other two underwent conservative treatment. During the follow-up, nine patients depicted progression of suprasellar lesions. Naylor et al. recommended that surgical debulking to obtain tissue for diagnosis and to open the CSF pathways was appropriate in managing suprasellar DIA/DIG (2). However, we hold that the maximal tumor resection should be attempted in all the patients because of the high residual tumor recurrence rate, but damaging the surrounding structures was unnecessary. Another notable finding in present study was that two patients showed spontaneous regression of the residual tumor, suggesting this possibility should be considered postoperatively.

Several adjuvant therapies, including vincristine, carboplatin, temozolomide, methotrexate, and etoposide, have been employed in the management of DIA/DIG (11–13). However, these interventions yielded disappointing outcomes. Notably, recent investigations have identified BRAF gene mutations in a subset of DIA/DIG cases, occurring at a frequency of approximately 43.8% (20). In our own study, 1 out of 3 suprasellar DIA/DIG tested positive for BRAF-V600E. Encouragingly, Blessing et al. successfully employed BRAF-MEK inhibitors, specifically dabrafenib and trametinib, in the treatment of an infantile DIG presenting with leptomeningeal brainstem dissemination, this treatment approach resulted in a reduction of both residual tumor size and leptomeningeal disease burden (21). This observation hints at the promising therapeutic potential of selective BRAF inhibitors in a subset of DIA/DIG with BRAF gene mutation. The application of radiation in suprasellar DIA/DIG is controversial. Recently, Wang et al. reported 18 in 188 DIA/DIG patients received radiation therapy and they found radiation therapy was not a predictor of mortality and tumor recurrence/progression based on a systematic literature review (22). Song et al. reported that 4 of 16 patients with gangliogliomas treated with GK radiosurgery showed no recurrence after a long period (23). Naylor et al. reported a suprasellar DIA/DIG patient received chemotherapy and whole-brain radiation therapy after partial tumor resection, and the patient is living 9 years after the initial diagnosis (2). Hence, further larger sample studies are needed to evaluate the use of radiation in treating DIA/DIG, especially for the patients with residual or recurrent tumor.

### Limitations

Due to the retrospective nature of the study and the involvement of rare cases collected over an extended time span, some extensive molecular testing of the tumor based on the 2021 World Health Organization (WHO) classification of brain tumors was lacking, and future studies were required to address the issue. Besides, we acknowledge that the sample size in this study is relatively small. Although the findings provide valuable insights and reference value for clinical practice, the conclusions should be interpreted with caution and require further validation through larger, multicenter cohorts.

## Conclusion

We summarized the clinical characteristics and outcomes of suprasellar DIA/DIGs based on the 5 institutional cases and a literature review. Our findings reveal these lesions typically manifest as solid masses characterized by a highly vascularized structure and strict adhesion to the surrounding tissue. Despite their classification as WHO Grade I tumors, suprasellar DIA/DIG exhibit a generally unfavorable prognosis, marked by a high rate of residual tumor recurrence, reaching the half. Notably, they display a heightened incidence of multifocal involvement, affecting up to 8/18 of cases. Besides the suprasellar region, their presence may extend to intraventricular, posterior fossa, and spinal cord locations. We highlight DIA/DIG should be included in the differential diagnosis of suprasellar masses of infancy. Long-term close follow-up is necessary due to the multiple locations and high tumor recurrence rate.

## Data Availability

The original contributions presented in the study are included in the article/**Supplementary Material**. Further inquiries can be directed to the corresponding author/s.
