# Supplementary material for: Suprasellar desmoplastic infantile astrocytoma and ganglioglioma: an institutional series report and a clinical summary of this rare tumor
Source: Front Oncol. 2025 Dec 10;15:1674829. doi: 10.3389/fonc.2025.1674829 (PMC12727624; doi:10.3389/fonc.2025.1674829)
Supplement: Supplementary file 1 [file Table1.docx]

| Authors | Age(months) | Sex(M/F) | Symptoms | Location | Texture | Extent of surgery | Adjuvant therapy | Follow-up | Recurrence/progression | Outcome |
| --- | --- | --- | --- | --- | --- | --- | --- | --- | --- | --- |
| Blessing et al,2018 | 4 | Female | Emesis; Hydrocephalus | Optic pathway/suprasellar | Solid-cystic | Subtotal | Chemotherapy (Carboplatin, cyclophosphamide, and vincristine) | 139 | Yes | Alive |
|  | 2 | Male | Enlarging head circumference; Brisk reflexes | Left frontal, basal ganglia, and hypothalamus | Solid-cystic | Subtotal | No | 83 | No | Alive |
| Naylor et al,2018 | 3 | Female | Rapidly enlarging head circumference nystagmus | Suprasellar | Solid | Partial | Chemo & radiation(unknown) | 108 | Yes | Alive |
|  | 2 | Male | Downward gaze, rapidly enlarging head circumference | Suprasellar | Solid | Partial | No | 60 | No | Alive |
|  | 4 | Male | Rapidly enlarging head circumference, nystagmus | Suprasellar | Solid | Partial | No | 4 | Yes | Dead |
| Lönrot et al,2007 | 5 | Male | Seizures, Increase in head circumference | Suprasellar, intraventricular, prepontine cistern | Solid | Partial | No | 24 | No | Alive |
| Jurkiewicz et al,2015 | 4.8 | Male | Simple partial seizure, myoclonic seizures | Temporal, left cerebral peduncle suprasellar cistern, interpeduncular cistern, prepontine cistern | Solid-cystic | Subtotal | No | 21 | Yes | Alive |
|  | 9 | Female | Nystagmus | Temporal right suprasellar cistern/area, optic tract L&R basal cisterns | Solid-cystic | Subtotal | Carboplatin and vincristine | 58 | No | Alive |
| Setty et al, 1997 | 4 | Male | Macrocephaly, Nystagmus | Suprasellar region; hypothalamus; posterior fossa; spinal canal | Solid | Biopsy | Chemotherapy(unknown) | 38 | NA | Alive |
| Bock et al, 2002 | 4 | Male | Increased intracranial ICP | Suprasellar region; diffuse leptomeningeal spread | Solid | Biopsy | Vincristine, cyclophosphamide, methotrexate, carboplatine, and etoposide | 5 | Yes | Dead |
| De Munnynck,2002 | 24 | Female | Macrocephaly and vomiting | Right hemisphere, hypothalamic mass, pial and the ependymal | Solid | Gross total(Right hemisphere） | Vincristine and carboplatinum | 11 | New occurrence of hypothalamic mass as well as of the pial and the ependymal metastases | Dead |
| Darwish et al, 2007 | 4 | Male | Irritability, failure to thrive and increasing head circumference | Suprasellar region; outlet of the fourth ventricle | Solid | Partial excision, VP | Vincristine and carboplatin | 0.25 | Suprasellar mass progressed, Multiple new enhancing nodules depicted on the tentorium and brain stem | Dead |
| Abuharbid et al, 2015 | 11 | Female | Nystagmus | Suprasellar region, cerebellar vermis，spinal cord | Solid | Gross total(cerebellar vermis） | No | 16 | Suprasellar tumor progressed, Spinal cord tumor regressed | Alive |
| Our series | 6 | Male | Nystagmus | Suprasellar cistern, bilateral cavernous sinus and hippocampus, intraorbital and cisterna magna | Solid | Partial | No | 26 | Suprasellar tumor regressed, lesion of fourth ventricle outlet disappeared | Alive |
|  | 85 | Female | Nystagmus | Suprasellar region | Solid | Subtotal | Temozolomide | 60 | Stable | Alive |
|  | 12 | Male | Downward gaze | Suprasellar region | Solid | Partial | No | 15 | Suprasellar tumor progressed | Dead |
|  | 1 | Female | Seizures | Suprasellar region | Solid | Subtotal | No | 24 | Stable | Alive |
|  | 2 | Female | Seizures | Suprasellar region | Solid | Partial | No | 41 | Tumor regressed | Alive |

Supplement table 1. Previously described patients with suprasellar DIA/DIGs and our institutional series.
